# Interplay between endoplasmic reticulum stress and non-coding RNAs in cancer

**DOI:** 10.1186/s13045-020-01002-0

**Published:** 2020-12-02

**Authors:** Tianming Zhao, Juan Du, Hui Zeng

**Affiliations:** Department of Hematology, The First Affiliated Hospital of Jinan University, Guangzhou, 510632 Guangdong China

**Keywords:** ER stress, UPR, ncRNAs, Cancer, Interplay

## Abstract

To survive, cancer cells are subjected to various internal and external adverse factors, including genetic mutations, hypoxia, nutritional deficiencies, and drug toxicity. All of these factors result in the accumulation of unfolded proteins in the endoplasmic reticulum, which leads to a condition termed endoplasmic reticulum stress (ER stress) and triggers the unfolded protein response (UPR). UPR downstream components strictly control transcription and translation reprogramming to ensure selective gene expression, including that of non-coding RNA (ncRNAs), to adapt to adverse environments. NcRNAs, including microRNAs (miRNAs), long non-coding RNAs (lncRNAs), and circular RNAs (circRNAs), play important roles in regulating target gene expression and protein translation, and their aberrant expression is related to tumor development. Dysregulation of ncRNAs is involved in the regulation of various cellular characteristics of cancer cells, including growth, apoptosis, metastasis, angiogenesis, drug sensitivity, and tumor stem cell properties. Notably, ncRNAs and ER stress can regulate each other and collaborate to determine the fate of tumor cells. Therefore, investigating the interaction between ER stress and ncRNAs is crucial for developing effective cancer treatment and prevention strategies. In this review, we summarize the ER stress-triggered UPR signaling pathways involved in carcinogenesis followed by the mutual regulation of ER stress and ncRNAs in cancer, which provide further insights into the understanding of tumorigenesis and therapeutic strategies.

## Introduction

The endoplasmic reticulum (ER), a multifunctional organelle, is involved in regulating fundamental cellular processes, including nascent protein folding and modification, calcium storage, liquid biosynthesis, and detoxification. Unfavorable external and internal factors, such as hypoxia, nutrient deprivation, drug-induced toxicity, acidic extracellular pH, and genetic mutation, result in unfolded or misfolded protein accumulation in the ER lumen. Under these conditions, tumor cells trigger endoplasmic reticulum stress (ER stress) to reestablish intracellular homeostasis and promote cell survival. This self-adaptive response process is called the unfolded protein response (UPR), which aims to restore protein homeostasis [1, 2]. If these unfavorable factors persistently exist and cells fail to achieve self-adaptation, the ER-related apoptotic pathway is initiated [3, 4]. Briefly, there are three transmembrane sensor proteins located on the ER membrane involved in the UPR, including inositol-requiring enzyme 1 (IRE1), protein kinase RNA-like ER kinase (PERK), and activating transcription factor 6 (ATF6). In the absence of unfolded proteins, a 78 kDa glucose-regulated protein (GRP78, also known as Bip) binds to these sensor proteins and lock them in an inactive state. Once ER stress occurs, GRP78 dissociates from the ER membrane enzymes, resulting in their activation, and initiates the downstream UPR signaling pathway [5, 6].

Non-coding RNAs (ncRNAs) are abundant RNA transcripts without protein-coding potential that play an important role in the biological regulation process. It is reported that approximately 75% of the human genome is transcribed into ncRNAs based on the data in the Encyclopedia of DNA elements (ENCODE) project [7, 8]. MicroRNAs (miRNAs), long non-coding RNAs (lncRNAs), and circular RNAs (circRNAs) are the three important ncRNA species. MiRNAs are small RNAs with a length of 19–24 nucleotides, which inhibit translation or induce the degradation of messenger RNA (mRNA) by binding to the 3′-untranslated region (UTR) of target mRNA [9]. LncRNAs are greater than 200 nucleotides in length. They function to regulate the expression of some genes, form sponges with miRNAs, bind with RNA-binding proteins to reach regulatory sites, and be a central platform for assembling other molecules [10]. CircRNAs are single-stranded closed non-coding RNA molecules whose structure and function have been extensively studied in recent years [11]. The ncRNAs exert a wide range of biological regulatory functions, such as modulation of transcription, controlling the synthesis of specific proteins, and binding with specific regions of DNA to activate or inhibit basic processes of gene regulation [12]. Accumulating evidence has indicated that ncRNAs are dysregulated in tumors and involved in the processes of tumor initiation, metastasis, and drug resistance [13–16]. Indeed, alterations in ncRNAs processing in cancer are commonly reported, and potential mechanisms of the ncRNAs subtypes involved in tumorigenesis have been explored. Tumorigenesis can be controlled by either a single ncRNA or an interconnected regulatory network controlled by multiple ncRNAs [17–19]. Findings about ncRNAs are being actively translated into clinical practice. Some miRNAs that are stable in the blood could be the basis for accurate and sensitive screening for major cancers [20]. Clinical trials with drugs based on miRNAs have been initiated for different diseases [21]. In addition, some ncRNAs have been reported as biomarkers for the diagnosis and prognosis of disease or as novel therapeutic targets for cancer intervention [22–24].

Recently, a close functional relationship between ER stress and ncRNAs, including miRNAs, lncRNAs and circRNAs, has been reported. Cross-talk between ER stress and ncRNAs has been reported in cancer development, and determining the nature of this connection has important implications for developing effective strategies for controlling tumors. Intriguingly, certain specific ncRNAs could regulate the UPR signaling pathway, and UPR downstream components in turn bind to the promoter region of ncRNAs to promote their transcription [25, 26]. In this review, we summarize the ER stress-triggered UPR signaling pathways involved in carcinogenesis and discussed the mutual regulation of ER stress and ncRNAs (miRNAs, lncRNAs, and circRNAs) in cancer.

## Three UPR signaling pathways are involved in carcinogenesis

**Fig. 1:**
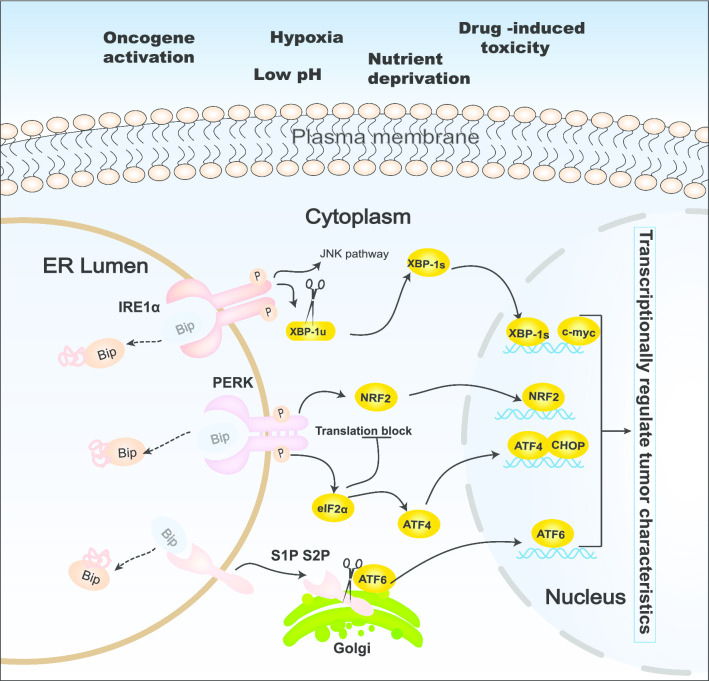
Role of the unfolded protein response (UPR) in cancer. Cancer cells frequently encounter intrinsic and extrinsic stresses that disturb protein folding in the endoplasmic reticulum (ER), including oncogene activation, hypoxia, nutrient deprivation, drug-induced toxicity, and low pH, which trigger ER stress to reestablish intracellular homeostasis. Upon detecting an accumulation of ER unfolded and misfolded proteins, the UPR is initiated by three transmembrane ER proteins: inositol-requiring enzyme 1(IRE1), protein kinase RNA-like ER kinase (PERK), and activating transcription factor 6 (ATF6). Once ER stress occurs, Bip dissociates from these three ER membrane enzymes, resulting in their activation and initiating the relevant downstream signaling pathway. Activation of the UPR can transcriptionally regulate tumor characteristics

To remain in a highly proliferative state, cancerous cells consume a great deal of substances, and these cells often perform aerobic glycolysis to support malignant expansion and develop a unique cancer microenvironment [27]. However, tumor cells can adapt to this harsh microenvironment by initiating ER stress. The UPR has three branched pathways, including the IRE1α, PERK, and ATF6 pathways (Fig. 1). Hyperactivation of these pathways have been reported to be involved in a wide range of human hematopoietic and solid tumors [28, 29].

### IRE1α-XBP1 pathway

IRE1α is the first specific sensor found to initiate ER stress, with serine and threonine protein kinase activity at its N-terminus and RNA endonuclease activity at its C-terminus [30]. When ER stress occurs, dimeric IRE-1 (IRE-1α and IRE-1β) undergoes conformational changes, resulting in autophosphorylation of IRE1 and activation of the IRE-1α endonuclease. Active IRE1α cleaves a 26-nucleotide intron from X-box binding protein-1 (XBP1) mRNA [31]. This spliced XBP1 (XBP1s) binds to various stress response gene promoters, which inhibits the expression of stress response proteins, upregulates the expression of proteins involved in protein folding and degradation, and promotes the correct folding of unfolded proteins and degradation of misfolded proteins, termed endoplasmic reticulum-associated degradation (ERAD) [30]. Activated IRE1α also interacts with TNF receptor-associated factor 2 (TRAF2) to activate downstream apoptosis signal-regulating kinase 1 (ASK1) and c-JUN amino-terminal kinase (JNK), resulting in increased apoptosis under prolonged UPR signaling [32, 33]. C-JUN may be a potential target for the design of antitumor drugs [34].

The IRE1α-XBP1s pathway is a highly evolutionarily conserved signaling pathway and is activated in tumor and immune cells [35–39]. RNA sequencing analysis revealed that IRE1α-XBP1s pathway activity is required for MYC signaling, which is a central oncogenic regulatory pathway in prostate cancer [40], building a direct connection between the UPR and oncogene activation. XBP1 promoted natural killer (NK) cell expansion in part by directly binding and activating the MYC promoter, which subsequently upregulated key MYC target genes required for NK cell expansion [39]. This novel IRE1α/XBP1/MYC axis in NK cells provided a new insight for host protection against NK cell-sensitive cancer. Interestingly, MYC transcriptionally regulates the expression of IRE1 in breast cancer [41]. This means that the IRE1-XBP1 signaling interacts with the presence of MYC hyperactivation. High expression levels of XBP1 are significantly associated with poor outcomes in human tumors, including prostate cancer [40], oral squamous cell carcinoma (OSCC) [42], hepatocellular carcinoma (HCC) [43], osteosarcoma [44], myeloma [45]. However, bortezomib (a proteasome inhibitor) is more effective in patients with high XBP1 expression. High XBP1 suggests a better prognosis in bortezomib-treated multiple myeloma [46]. Nonetheless, XBP1 promotes the progression of triple-negative breast cancer (TNBC) through synergy with hypoxia inducible factor-1 alpha (HIF1α) to support tumor-initiating cell function and the metastatic ability of cancer cells under adverse environmental conditions [35]. Inhibition of IRE1-XBP1 signaling may suppress tumor initiation, progression, and metastasis and overcome drug resistance [35, 47].

### PERK-eIF2α pathway

Under physiological conditions, the transmembrane protein PERK binds to Bip and maintains an inactive state. PERK is activated upon dissociation from Bip, resulting in phosphorylation of eukaryotic initiation factor 2α (eIF2α), which is essential for reducing the protein load in the endoplasmic reticulum [48]. At the same time, activation of PERK also upregulates the translation of activating transcription factor 4 (ATF4) mRNA, a member of the CCAAT/enhancer-binding protein (C/EBP) family that induces the increased expression of protein transport-related genes after entering the nucleus [48]. ATF4 also activates the transcription of C/EBP homologous proteins (CHOP). A combination of ATF4 and CHOP upregulate protein transcription of growth arrest and DNA damage-inducible protein 34 (GADD34), which in turn leads to dephosphorylation of eIF2α to restore the expression of protective proteins. Notably, activation of the ATF4-CHOP induces the apoptotic pathway when cell damage exceeds the UPR processing capacity [49, 50]. In addition, eIF2α induces abnormal activation of nuclear factor-κB (NF-κB) to inhibit the expression of apoptosis-related proteins [51]. PERK activation rapidly and directly phosphorylates nuclear factor-erythroid 2-related factor 2 (NRF2) to promote cell survival and induce resistance to ER stress and chemosensitivity [52, 53].

A series of stressful conditions that are unfavorable for tumor growth may trigger disruption of ER homeostasis and lead to ER stress, further activating the PERK-eIF2α branch of the UPR, which may contribute tumor cells adapting to harsh environmental conditions. For example, under low glucose metabolism stress, PERK activation induces glioma cell survival through AKT activation. PERK-silenced glioma cells show decreased tumor formation capacity [54]. The PERK/eIF2α branch and its downstream components also play a pivotal role in regulating autophagy to promote cancer cell survival. Moreover, in cancer cells, hypoxia can upregulate PERK to induce the expression of autophagy-related molecules, indicating the role of PERK in promoting autophagy and cancer development [55]. Furthermore, MYC expression is associated with a sharp increase in PERK activity in human lymphoma. MYC-expressing cells appear to be completely reliant on PERK-dependent autophagy [56]. PERK activation has been also implicated in tumor invasiveness. It has been confirmed that PERK is involved in distant breast cancer metastasis via regulation of the downstream mediator CREB3L1 [57]. In addition, overexpression of ATF4, a downstream molecule in the PERK pathway, stimulated the expression of matrix metalloproteinases (MMPs) MMP2 and MMP7 to induce invasion and metastasis in esophageal squamous cell carcinoma [58]. Moreover, current evidence suggests that forkhead box O class protein 3 (FOXO3) can directly regulate PERK expression. Deletion of FOXO3 significantly reduced PERK expression and enhanced sensitivity to a PERK inhibitor in breast cancer [59].

### ATF6α pathway

ATF6, a member of the leucine zipper transcription factor family, is a transmembrane protein on the endoplasmic reticulum that is an important regulator involved in apoptosis and autophagy in ER stress [60]. ATF6 has two homologs, ATF6α and ATF6β, which are expressed in mammalian cells. Upon ER stress, ATF6α is transferred to the Golgi and processed by site-1 protease (S1P), and S2P to generate cleaved ATF6α, its active form. This active ATF6α acts as a transcription factor into the nucleus and regulates gene expression by the ATF-cAMP response element or ER stress response element (ERSE). In addition, ATF6α can also bind to UPR elements, activating CHOP [61, 62].

Due to the major pro-survival role of ATF6, its expression level has been shown to be significantly upregulated in various cancer types [63–66]. Higher expression level of ATF6 has been closely correlated with cancer metastasis and recurrence [67, 68] and served as a prognostic indicator of cancer [69]. For instance, ER stress-related ATF6 upregulated cancerous inhibitor of protein phosphatase 2A (CIP2A), which contributes to colon cancer cell survival and indicates a trend toward poor prognosis [69]. In addition, ATF6 has also been shown to confer poorer response to chemotherapy. Knockdown of ATF6 or pharmacological inhibition of its downstream targets have better sensitivity to chemotherapy [70, 71]. Missense mutations in TP53 enhance ATF6 activity and coordinate with inhibition of the pro-apoptotic factors JNK and CHOP, which are necessary for viability and invasion [72]. Overall, ATF6 plays a crucial role in promoting tumor progression and may be a promising therapeutic target, although no specific inhibitors have been identified yet.

## ER stress regulates ncRNAs expression

It has been reported that ER stress affects the expression of miRNAs and lncRNAs, but there have been no reports on circRNAs. Therefore, whether and how ER stress regulate circRNAs expression need to be further investigated.

### ER stress influences miRNAs expression

**Fig. 2:**
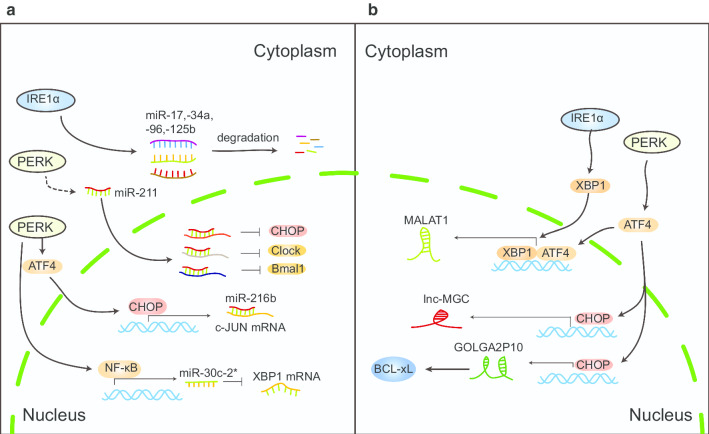
ER stress regulates miRNAs and lncRNAs expression. **a** Sustained IRE1α RNase resulted in rapid degradation of miR-17, miR-34a, miR-96, and miR-125b. PERK-induced miR-211 inhibits CHOP, Bmal1, and Clock in tumor cells. PERK promotes translocation of NF-κB into the nucleus to control the expression of miR-30c-2* under ER stress. miR-30c-2* in turn negatively regulates the expression of XBP1s mRNA. **b** Activated IRE1 and PERK signaling pathways increase the MALAT1 expression. The transcription factor CHOP transcriptionally regulates expression of lnc-MGC and GOLGA2P10

The expression of several miRNAs has been demonstrated to be altered by ER stress in response to adverse conditions (Table 1, Fig. 2a). Wang et al. reported that the expression levels of miR-214, miR-199a-3p, and miR-199a-5p were significantly reduced in HCC cells treated with an ER stress inducer and exposure to anoxia. Low expression of miR-214 relieves the inhibitory effect on tumor cells, which was shown to promote tumor cell progression [73]. Furthermore, restoring the level of miR-199a-3p in HCC cells led to G1 phase cell cycle arrest, reduced invasiveness, increased sensitivity to hypoxia, and increased sensitivity to doxorubicin-induced apoptosis through targeting rapamycin (mTOR) [74]. Jiang et al. revealed that increased miR-1281 induced by ER stress promoted apoptosis in osteosarcoma. p53 directly bound to the promoter of miR-1281, contributing to its transcription under ER stress. Luciferase reporter gene assays showed that USP39 was the target of miR-1281 [75].

**Table 1:** ER stress-regulated miRNAs and their potential roles in cancers

Effectors	Regulators	Targets	Cancer type	Biological process	References
miR-214	NA	XBP-1	HCC	Regulates HCC cell proliferation and apoptosis	[73]
miR-199a-3p/5p	NA	NA	HCC	NA	[73]
miR-1281	NA	USP93	Osteosarcoma	Promotes ER stress-mediated apoptosis	[75]
miR-34a	IRE1α	MYC, cyclin D1, CDK4	AML	Inhibition of IRE1α increases expression of miR-34a in AML cells	[78]
miR-216b	PERK	c-Jun	Osteosarcoma	Sensitizes cells to ER stress-dependent apoptosis	[79]
miR-211	PERK	CHOP	Osteosarcoma	Inhibits circadian rhythm oscillations and ongoing protein synthesis	[81]
miR-211	PERK	CHOP	Mammary tumors	Regulates ER stress-dependent apoptosis	[84]
miR-663	NA	ZBTB7A	Osteosarcoma	Regulates ER stress-induced cell apoptosis	[88]
miR-23a-3p	NA	PTEN AKT	HCC	Upregulates macrophage PD-L1 expression and inhibits T cell function, which promotes tumor cells to escape immune surveillance	[115]
miR-765	NA	FOXA2	Melanoma	Enhances tumor stem cell renewal and apoptosis inhibition	[120]
miR-663	NA	TGFB1	HCC	Regulates ER stress-induced apoptosis	[184]
miR-221/222	NA	NA	HCC	Promotes ER stress-mediated apoptosis	[185]
miR-346	XBP1	TAP1	Cervical cancer	Reduces MHC class I-associated antigen presentation	[186]

NA, not available; HCC, hepatocellular carcinoma; AML, acute myeloid leukemia

IRE1 has endoribonuclease (RNase) activity, which can remove a 26 base pair intron from XBP1 mRNA to generate activated XBP1s. XBP1s stimulates the synthesis of multiple UPR target genes [76]. Sustained IRE1α RNase activation results in rapid degradation of certain miRNAs, including miR-17, miR-34a, miR-96, and miR-125b, which normally inhibit caspase-2 mRNA translation, leading to a sharply increasing protein level of this initiator protease in the apoptotic pathway [77]. On the contrary, inhibition of IRE1α RNase activity through the use of small-molecule inhibitors (2-hydroxy-1-naphthaldehyde, HNA) can increase the expression of many miRNAs in acute myeloid leukemia (AML) cells, including miR-34a. MiR-34a, in turn, enhances the sensitivity of AML cell lines to IRE1 inhibitors [78]. Therefore, inhibiting the IRE1α-driven survival pathway may be a promising application for the treatment of AML.

PERK was previously thought to be a regulator of miRNAs accumulation during ER stress. Indeed, ER stress-induced miRNAs expression partially depends on the PERK-eIF2α-ATF4-CHOP pathway [79–81]. CHOP also regulates miRNAs expression under ER stress in tumor cells. Studies have demonstrated that CHOP binds to the miR-216b promoter region and regulates miR-216b expression in a Dicer-dependent manner during ER stress. miR-216b directly binds to the 3′ UTR of c-JUN, antagonizes c-JUN accumulation and thereby enhances apoptosis [79, 82]. In addition, PERK could activate NF-κB via phosphorylation of eIF2α, which translocate into the nucleus to activate target genes [83]. For example, NF-κB, as a downstream molecule of PERK, is involved in inducing the regulation of miR-30c-2* expression under ER stress. miR-30c-2* in turn negatively regulates the mRNA expression of XBP1s [80]. Previous studies have demonstrated that PERK signaling induces expression of miR-211, which directly targets the proximal CHOP promoter, where it increases histone methylation and inhibits CHOP expression. In other words, miR-211 is a pro-survival miRNA that regulates CHOP expression in a PERK-dependent manner [84]. PERK-induced miR-211 inhibition of Bmal1 and Clock in tumor cells is another mechanism that has been recently demonstrated where this pathway inhibits circadian rhythm oscillations and ongoing protein synthesis, thereby promoting tumor progression [81].

### ER stress regulates lncRNAs expression

In response to ER stress, tumor cells inevitably regulate a variety of gene expression levels, including that of long non-coding RNAs. Recently, it has been found that lncRNAs expression can be regulated by ER stress to participate in the regulation of survival and migration of cancer (Table 2, Fig. 2b). Some UPR downstream transcription factors regulate lncRNAs transcription. For instance, the transcription factor CHOP has been reported to be a key transcriptional regulator to control lnc-MGC. Knockdown of CHOP using siRNA significantly suppressed the induction of lnc-MGC [85]. Wu et al. reported that CHOP can directly bind to the promoter of the lncRNA Golgin A2 pseudogene 10 (GOLGA2P10). Aberrant expression of GOLGA2P10 increases the level of the anti-apoptosis gene BCL-xL, which confers cancer cells with resistance to the cytotoxic effects of ER stress and are more likely to survive under harsh conditions [26]. In addition, lncRNAs metastasis-associated lung adenocarcinoma transcript 1 (MALAT1) is upregulated by a pharmacological agent of ER stress induction [86]. Further studies have shown that activated IRE1 and PERK signaling pathways increase the expression of MALAT1, which promote colorectal cancer (CRC) cell migration. Bioinformatic analysis has indicated XBP1 and ATF4 binding sites within the MALAT1 gene promoter region [87]. It has been believed that ER stress-regulated lncRNAs play critical roles in tumor progression. For instance, ABTB7A acts as an important pro-survival factor in osteosarcoma cells. Under pharmacological ER stress, osteosarcoma cells downregulate ABTB7A expression and promotes apoptosis. Further mechanistic studies revealed that miR-663a induced by ER stress directly binds to the 3′UTR of ZBTB7A and mediates ER stress-induced ZBTB7A downregulation. Interestingly, ABTB7A transcriptionally inhibited the expression of lncRNA GAS5 by directly binding the promoter of lncRNA GAS5 [88].

**Table 2:** ER stress-regulated lncRNAs and their potential mechanisms in cancers

LncRNAs	Expression level	Tumor type	UPR-related mechanism	Biological process	References
GOLGA2P10	Upregulated	HCC	CHOP can directly bind to the GOLGA2P10 promoter	Induces resistance to cytotoxic effect of ER stress	[26]
MALAT1	Upregulated	Colorectal cancer	Activated IRE1 and PERK signaling pathways increase MALAT1expression	Promotes the migration of CRC cells	[87]
MIAT	Upregulated	Breast cancer	Overexpression of GRP78 upregulates the expression of MIAT by increasing OCT4 in 5-fluorouracil resistant cells	Contributes to 5-FU resistance	[175]

HCC, hepatocellular carcinoma

## NcRNAs regulate UPR in cancer progression

### MiRNAs regulate UPR in cancer

**Fig. 3:**
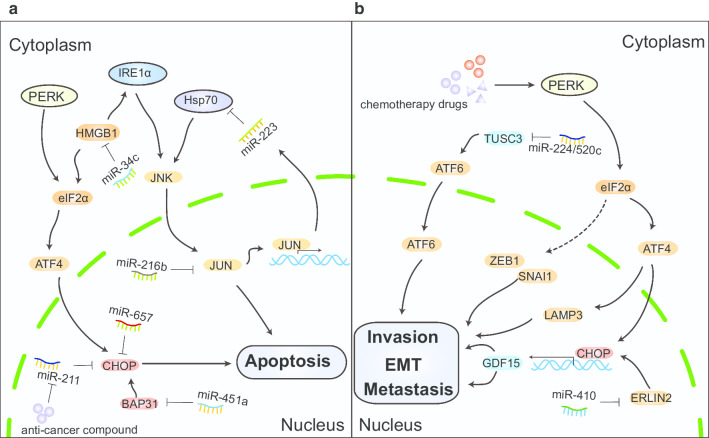
MiRNAs regulate tumor characteristics including apoptosis, invasion, EMT, and metastasis by regulating the UPR. **a** MiR-657 mimics can attenuate the CHOP expression to reverse apoptosis. Anticancer compounds downregulate the expression of miR-211 in U937 and U266 cells. The downregulated miR-211 is associated with CHOP and triggers tumor cell apoptosis. MiR-34c overexpression significantly increased the levels of eIF2α and IRE1α by directly targeting the 3ʹUTR of HMGB1 and inhibiting HMGB1 translation, promoting apoptosis. The expression of miR-216b directly targets c-JUN and inhibition of c-JUN sensitizes cells to apoptosis. MiR-451a increases apoptosis by suppressing BAP31 to induce ER stress. MiR-233 downregulates the heat shock protein 70 (Hsp70) protein levels and downstream JNK/JUN signaling pathways, thereby enhancing apoptosis. JUN can bind to the promoter region of miR-223 to promote its transcription, forming a feedback loop. **b** Some chemotherapy drugs activate the PERK pathway by upregulating the expression levels of SNAI1 and ZEB1. LAMP3 is regulated by activation of the PERK/eIF2a/ATF4 arm of the UPR to promote lymph node metastasis. CHOP induced by PERK- eIF2α can bind to GDF15 and activate its transcription, regulating EMT and metastasis. MiR-410 directly targets ERLIN2 to up-regulate UPR components to inhibit the migration, invasion, and EMT of breast cancer cells. MiR-224/-520c-dependent TUSC3 deletion enhances NSCLC metastasis via increased ATF6α activity

MiRNAs directly or indirectly act on UPR pathway molecules to regulate intracellular homeostasis and affect carcinogenic processes, including survival, apoptosis, invasion, metastasis, cancer stem cell characteristics, and the tumor microenvironment (Table 3, Fig. 3).

**Table 3:** MiRNAs directly or indirectly regulate UPR pathway components

Regulators	Effectors	Targets	Cancer type	Biological process	Reference
miR-233	HSP70	HSPA1A	Osteosarcoma	Regulates apoptosis	[25]
miR-216b	CHOP	c-JUN	Osteosarcoma	Sensitizes cells to apoptosis	[79]
miR-30c-2*	XBP1	XBP1	Cervical cancer	Influences the fate of cells challenged with ER stress	[80]
miR-657	CHOP	NA	Hematological cancer	Attenuates the expression of CHOP, p-ATF2, and PARP cleavage to reverse SSD-induced apoptosis	[91]
miR-211	CHOP	NA	Lymphoma, multiple myeloma	Attenuates COM-induced apoptosis	[92]
miR-34c	eIF2α CHOP IRE1α	HMGB1	NSCLC	Inhibits cell proliferation, promotes apoptosis, and induces ER stress in NSCLC cells	[93]
miR-451a	GRP78 PERK elF2α ATF4 CHOP	BAP31	Colorectal cancer	Inhibits proliferation and increases apoptosis	[98]
miR-410	CHOP GRP94 GRP78 eIF2α	ERLIN2	Breast cancer	Inhibits cell migration and invasion and EMT	[108]
miR-224 miR-520c	ATF6α	TUSC3	NSCLC	Enhances UPR and ERAD to promote metastatic potential of	[109]
miR-122	GRP78 CHOP	CDK4	HCC	Regulates anticancer drug–mediated apoptosis	[167]
miR-146a	CHOP	CHOP	Lung cancer	Reduces the sensitivity of lung cancer cells to cisplatin	[171]
miR-7112-3p	PERK	PERK	Colorectal cancer	Enhances apoptosis in CX-1 cells treated with DVDMS-PDT	[174]
miR-1202	GRP78	Rab1A	Glioma	Inhibits proliferation and induces ER stress and apoptosis	[187]
miR-15b-5p	GRP78	Rab1A	HCC	Induces apoptosis	[188]
miR-1291	IRE1α	IRE1α	HCC	Regulates glypican-3 mRNA expression	[189]
miR-30d miR-181a miR-199a-5p	GRP78	GRP78	Prostate cancer	Suppresses GRP78 levels and GRP78-mediated chemoresistance	[190]

NA, not available; NSCLC, non-small cell lung cancer; HCC, hepatocellular carcinoma

#### Survival and apoptosis

CHOP activation has long been generally considered to be the main pathway for ER stress-related apoptosis [89]. A recent study has shown that the deficiency/deletion of CHOP in T cells triggers spontaneous antitumor CD8^+^ T cell activity and increases the effects of T cell immunotherapy [90]. Selected miRNAs act indirectly on CHOP to regulate tumor cell apoptosis. Treatment of hematological tumor cells with *spatholobus suberectus* Dunn (SSD) can upregulate ER stress-related proteins, including CHOP and p-ATF2, whereas miR-657 is significantly reduced. MiR-657 mimics can attenuate the expression of CHOP, p-ATF2, and PARP cleavage to reverse SSD-induced apoptosis [91]. Similarly, *Cnidium officinale* Makino (COM) has been known to be an anticancer compound that also downregulates the expression of miR-211 in U937 and U266 cells. The downregulated miR-211 is associated with CHOP and triggers tumor cell apoptosis [92]. Moreover, the overexpression of miR-34c, a tumor suppressor, significantly increased the levels of eIF2α and IRE1α by directly targeting the 3ʹUTR of HMGB1 and inhibits HMGB1 translation, promoting non-small cell lung cancer (NSCLC) apoptosis [93]. MiRNAs usually target mRNAs to cause translation inhibition and degradation. However, whether those miRNAs directly targeted CHOP mRNA requires further elucidation.

Under severe and irreparable stress conditions, the IRE1α-ASK1-JNK/c-JUN signaling pathway may trigger apoptosis [33, 94]. JNK downregulates anti-apoptotic proteins, such as BCL-2, BAD, and BAX, and simultaneously activates pro-apoptotic BID, BIM, and Bcl-2-modified factors (BMF) to initiate apoptosis [95, 96]. However, it should be noted that the UPR-mediated JNK signaling is biphasic. When it is immediately activated in its early stage, it has an anti-apoptotic effect, but in the late stage, it can promote cell death. This opposite effect of JNK on cell viability exists in ER stress [97]. Evidence suggests that ER stress-dependent miR-216b induction occurs via a pathway consisting of PERK, eIF2a, ATF4, and CHOP. The expression of miR-216b directly targets c-JUN, and inhibition of c-JUN sensitizes cells to apoptosis. CHOP-dependent miR-216b transcription downregulates c-JUN expression, thereby amplifying the pro-apoptotic activity of CHOP [79]. Similarly, miR-451a can regulate CRC cell survival by activating ER stress. Elevated miR-451a increases the expression of ER stress-associated proteins, including BIP and PERK/elF2α/ATF4/CHOP. Dual-luciferase reporter assays detected that B cell receptor-associated protein 31 (BAP31) was a direct target of miR-451a. MiR-451a inhibits proliferation and increases apoptosis by suppressing BAP31 to induce ER stress in CRC [98]. In addition, miR-233 downregulates the heat shock protein 70 (Hsp70) protein level and downstream JNK/JUN signaling pathways by binding to the HSPA1A 3′UTR, thereby regulating osteosarcoma cells apoptosis. JUN is a downstream transcription factor of JNK signaling and can bind to the promoter region of miR-223 to promote its transcription. In short, miR-223, Hsp70, and downstream JNK/JUN form a feedback loop [25] (Fig. 3a).

#### Invasion and metastasis

Carcinoma cells reprogram their differentiation status through the epithelial-to-mesenchymal transition (EMT), thereby acquiring the key malignant characteristics of invasion and metastasis. Current evidence suggests that UPR signaling promotes tumor progression through activation of the invasion-metastasis cascade, of which EMT plays a vital role [99]. In human tumor tissue, EMT gene expression is closely related to the extracellular matrix (ECM) and PERK-eIF2α signaling but not to other branches of the UPR [100]. Evidence suggests that some chemotherapy drugs such as cisplatin, cytarabine, doxorubicin, gemcitabine, vinorelbine, etoposide, and pemetrexed activate the PERK pathway and eventually induce EMT by upregulating the expression levels of SNAI1 and ZEB1 [101]. ER stress is often considered a drug-induced side effect caused by these anticancer drugs.

Hypoxia can not only act as a stressor to activate ER stress [102] but also as an inducer of EMT in cancer [103]. Lysosomal-associated membrane protein 3 (LAMP3), a hypoxia-inducible gene, is regulated by activation of the PERK/eIF2a/ATF4 arm of the UPR to promote lymph node metastasis in breast and cervical cancer [104, 105]. Interestingly, under hypoxia exposure, CHOP induced by PERK-eIF2α can bind to growth differentiation factor 15 (GDF15) and activate its transcription, regulating EMT and the metastasis of colorectal cancer cells. This indicates that CHOP-activated GDF15 expression is required to maintain CRC cell survival [106]. IRE1 has also been involved in promoting cell survival under hypoxic conditions, and wild-type cells exposed to hypoxia have reduced in vitro survival compared to XBP1-deficient cells [107]. It has been reported that cancer cells undergoing EMT adapt to ER stress by activating the PERK branch of the UPR. Disruption of the PERK pathway significantly increases the sensitivity of cancer cells to ER stressors [100]. MiRNAs regulate ER stress by acting on target genes, which plays an important role in EMT, promoting tumor invasion and metastasis. MiR-410 acts as a tumor suppressor to inhibit cell migration, invasion, and EMT in breast cancer cells. Further studies have shown that miR-410 can enhance the levels of CHOP, GRP94, Bip, and p-PERK. Endoplasmic reticulum lipid raft-associated 2 (ERLIN2) is a direct target of miR-410 [108]. MiR-224/-520c-dependent TUSC3 downregulation enhances the metastasis of NSCLC through increased ATF6α activity [109] (Fig. 3b).

#### Tumor microenvironment

**Fig. 4:**
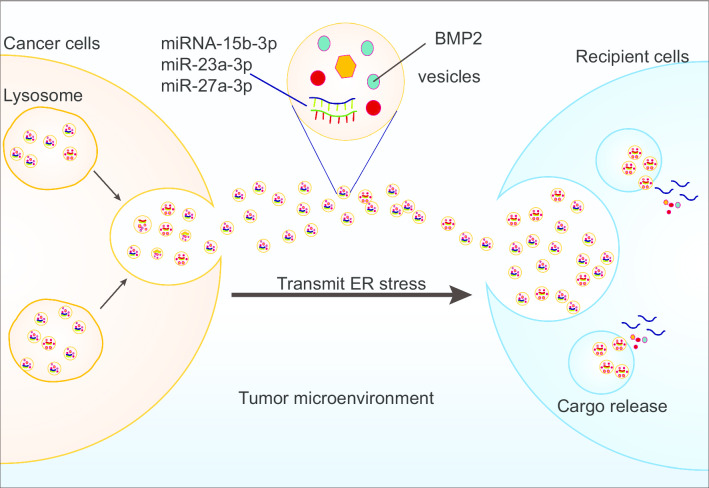
ER stress is transmitted by extracellular vesicles harboring miRNAs in the tumor microenvironment. Extracellular vesicles derived from tumor cells carry miRNAs and/or proteins that transmit ER stress from tumor cells to recipient cells, thus affecting the function of recipient cells

Tumor cells with high metabolism are prone to hypoxia, glucose deficiency, lactic acidosis, oxidative stress, and reduced amino acid supply. All of these changes in the microenvironment contribute to activation of the UPR [3, 110]. Cancer cells are exposed to ER stress secrete unknown soluble factors, and these mediators can cause macrophages to initiate ER stress accompanied by transcriptional activation and pro-tumor proinflammatory cytokine secretion in a toll like receptor 4 (TLR4)-dependent manner [111]. Similar studies have also demonstrated that cytokines in the tumor microenvironment, such as IL-4, IL-6, and IL-10, can activate the IRE1α-XBP1 branch [37]. Extracellular vesicles, particularly exosomes, as an important component in the tumor microenvironment, can also be used as a medium for transmitting ER stress. A current study has shown that extracellular vesicles derived from AML cells carry BMP2 to transmit ER stress to mesenchymal stem cells (MSCs) and osteoblastic progenitor cells (OPCs) [112]. MiRNAs are one of the most significant components in exosomes, which play an important role in the transmission of information between cells. For example, exosomes secreted by gastric cancer cells transfer miR-15b-3p to recipient gastric cancer cells, promoting the progression of gastric cancer through the dynein light chain Tctex-type 1/caspase-3/caspase-9 signaling pathway [113]. Transmissible ER stress also impacts the function of immune cells and subsequently promotes tumor survival, progression, and metastasis [111, 114]. ER-stressed HCC releases exosomes trafficking miR-23a-3p to upregulate PD-L1 expression in macrophages and inhibit T cell function, which promotes tumor cells to escape immune surveillance [115]. Similarly, ER stress contributes to exosome secretion and enhanced exosomal miR-27a-3p expression in breast cancer. Exosomes carrying miR-27a-3p target macrophages in the microenvironment. MiR-27a-3p could target MAGI2 and negatively regulate MAGI2 expression, while downregulation of MAGI2 upregulated PD-L1 expression via the PTEN/PI3K signaling pathway [66]. In general, in the tumor microenvironment, ER-stressed tumor cells transmit information to other cells, such as immune cells, through exosomes carrying cargo (Fig. 4).

#### Cancer stem cell characteristics

Tumor stem cells can remain dormant for a long period of time and are insensitive to a variety of unfavorable risk factors, which play critical roles in tumor initiation, progression, recurrence, and metastasis [116, 117]. It has been shown that tumor stem cells exhibit enhancement for ER stress resistance, which contributes to tumor growth, angiogenesis, and resistance to chemotherapy [118, 119]. In ER stress-resistant melanoma, the number of cancer stem cells is significantly increased. The underlying mechanism involves Hoxb9 binding to the miR-765 promoter and promoting its transcription. The highly expressed miR-765 targets Forkhead box A2 (Foxa2), resulting in a decrease in Foxa2 expression, and enhancement of tumor stem cells renewal, and apoptosis inhibition [120]. Further research is needed to eliminate tumor stem cells by regulating ER stress.

### LncRNAs regulate the UPR in cancer progression

**Fig. 5:**
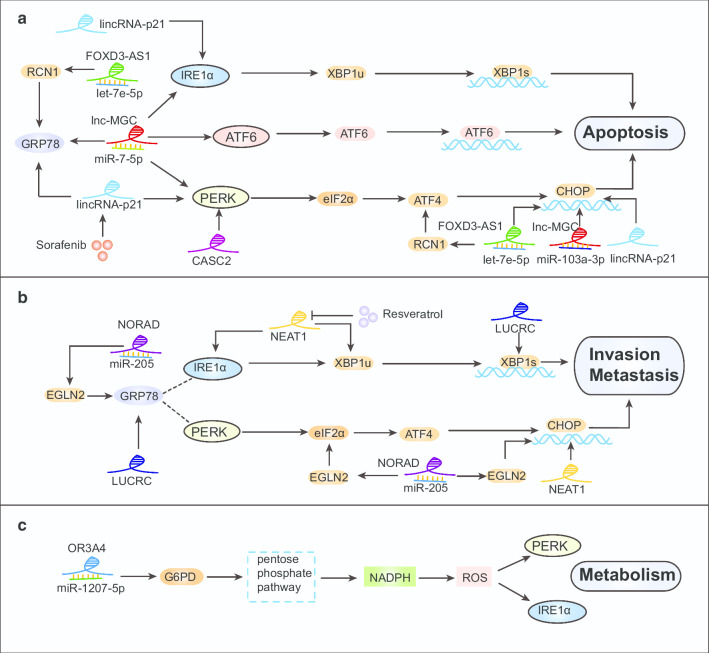
LncRNAs regulate the tumor characteristics apoptosis (**a**), invasion and metastasis (**b**), and metabolism (**c**) by regulating the UPR. **a** FOXD3-AS1 could competitively bind to let-7e-5p to regulate RCN1. Silencing FOXD3-AS1 or upregulating let-7e-5p increased the expression profiles of GRP78, CHOP, and ATF4, consequently promoting ER stress-induced apoptosis. MEG3 increased the expression of ER stress-related proteins, including GRP78, IRE1, PERK, ATF6, and CHOP, consequently inhibiting growth and induce the apoptosis of cancer cells. In addition, MEG3 competitively combines with miR-7-5p or miR-103a-3p to promote ER stress-mediated apoptosis. Ectopic expression of lincRNA-p21 activates ER stress by inducing the expression of IRE1, CHOP, and GRP78 and upregulating the PERK phosphorylation level. Sorafenib also could increase the expression of lincRNA-p21 to induce ER stress-mediated apoptosis. Overexpression of CASC2 increases the stability of PERK mRNA, which triggers the PERK/eIF2α/CHOP pathway and promotes radiation-induced apoptosis. **b** NORAD may act as a sponge for miR‐205 and reduce the transcriptional repression of the miR-205 target gene EGLN2. NORAD silencing can inhibit UPR-related gene expression including that of GRP78, CHOP, and eIF2α. Downregulation of NORAD also restrained malignant melanoma cell migration and invasion. Overexpression of NEAT1 promote expression of the UPR-related proteins IRE1, XBP-1, and CHOP. Resveratrol attenuates the effects of NEAT1 on inducing ER stress. LUCRC can regulate Bip expression and induce the splicing of XBP1 from XBP1u (unspliced) to XBP1s (spliced), resulting in activation of UPR to promote tumor progression. **c** OR3A4 functions as a sponge for miR-1207-5p, modulating the target gene glucose-6-phosphate dehydrogenase (G6PD). Knockdown of OR3A4 inhibits the expression of G6PD, blocks the pentose phosphate pathway, and significantly reduces the level of nicotinamide adenine dinucleotide phosphate (NADPH). This abnormal metabolic pathway upregulates the redox state content, which increases the expression of PERK and IRE1 in osteosarcoma cells

LncRNAs exert their molecular functions through RNA–protein, RNA-RNA, or RNA–DNA interactions. Abnormal lncRNAs expression has been implicated in cancer progression via their regulation of the UPR (Table 4, Fig. 5).

**Table 4:** LncRNAs regulate UPR pathway components

LncRNAs	Expression level	Tumor type	UPR-related mechanism	Biological process	References
MEG3		Breast cancer	Increases ER stress-related proteins (GRP78, IRE1, PERK, ATF6, and CHOP) and NF-κB	Inhibits growth, induces apoptosis	[121]
	Downregulated	ESCC	Increases ER stress-related proteins (GRP78, IRE1, PERK, ATF6, and CHOP), caspase-9, and cleaved caspase-3	Inhibits cell growth, induces apoptosis	[122]
	Downregulated	HCC	Increases ER stress-related proteins (GRP78, IRE1, PERK, ATF6, and CHOP)	Inhibits proliferation, induces apoptosis	[123]
	Downregulated	Cervical carcinoma	Competing endogenous RNA of miR-7-5p	Accelerate ER stress-mediated apoptosis	[124]
	Downregulated	Colorectal carcinoma	Increases ER stress-related proteins (GRP78, ATF6, and CHOP)	Suppresses proliferation and invasion	[125]
CASC2	Downregulated	NSCLC	Increases PERK mRNA stability	Promotes radiation-induced apoptosis	[127]
FOXD3-AS1	Upregulated	NPC	Silencing FOXD3-AS1 promotes ER stress-induced apoptosis by competitively binding to let-7e-5p	Silencing of FOXD3-AS1 induces apoptosis	[128]
LincRNA-p21	Downregulated	HCC	Induces expression of IRE1, CHOP, and GRP78 and upregulates the phosphorylation level of PERK	Contributes to sorafenib-induced ER stress and apoptosis	[132]
NORAD	Upregulated	Melanoma	Knockdown of NORAD can inhibit UPR-related genes including GRP78, CHOP, and eIF2α	Promotes invasion and migration	[136]
NEAT1	Upregulated	Multiple myeloma	Promotes expression of UPR-related proteins CHOP, XBP-1, and IRE1	Promotes proliferation, migration, and invasion	[141]
LUCRC	Upregulated	Colorectal cancer	Induces the expression of GRP78	Promotes proliferation, migration, and invasion	[142]
OR3A4	Upregulated	Osteosarcoma	Knockdown of OR3A4 inhibits the expression of G6PD, blocking the pentose phosphate pathway and resulting in ER-stress	Promotes proliferation, colony formation	[145]

ESCC, esophageal squamous cell carcinoma; HCC, hepatocellular carcinoma; NSCLC, non-small cell lung cancer; NPC, nasopharyngeal carcinoma

#### Apoptosis

Many tumor-related lncRNAs can regulate the proliferation and apoptosis of tumor cells by activating the UPR [121–123]. Recent studies have shown that ectopic expression of MEG3 increases the expression of ER stress-related proteins, including GRP78, IRE1, PERK, ATF6, and CHOP and is accompanied by NF‑κB translocation from the cytoplasm to the nucleus. Consequently, MEG3 may inhibit growth and induce the apoptosis of cancer cells [121–123]. It has been revealed that MEG3 competitively combines with miR-7-5p to upregulate the STC1 level, thus accelerating ER stress-mediated apoptosis in cervical carcinoma, which has been termed a competing endogenous RNA (ceRNA) model [124]. Moreover, MEG3 was downregulated in human CRC. Restoring MEG3 in these cells promote the expression of ER stress-related proteins, including GRP78, ATF6, and CHOP through the formation of a sponge with miR-103a-3p [125]. Another study has also shown that MEG3 remarkably upregulates the ER stress-related protein GRP78 and activates NF-κB signaling to affect the apoptosis of gallbladder cancer cells [126]. In addition, the lncRNA CASC2 was negatively correlated with the malignancy of NSCLC cells. Overexpression of CASC2 increases the stability of PERK mRNA, which triggers the PERK/eIF2α/CHOP pathway and promotes radiation-induced apoptosis of NSCLC cells [127]. FOXD3-AS1 has been shown to be upregulated in nasopharyngeal carcinoma tissues and cells. Further research has revealed that FOXD3-AS1 could competitively bind to let-7e-5p to regulate RCN1. Silencing FOXD3-AS1 or upregulating let-7e-5p increases the expression profiles of GRP78, CHOP, ATF4, caspase-12, and caspase-9, consequently promoting ER stress-induced apoptosis in nasopharyngeal carcinoma [128]. Furthermore, lincRNA-p21 acts as a tumor suppressor and is downregulated in numerous tumors. Overexpression of lincRNA-p21 significantly inhibits cell proliferation capacity, induces G1 arrest and apoptosis, and increases radiosensitivity of hepatocellular carcinoma cells [129, 130]. LincRNA-p21 is also involved in regulating drug resistance. Sorafenib, a small molecule antitumor drug, could induce the apoptosis of hepatocellular carcinoma cells by inducing ER stress [131]. Interestingly, sorafenib also could induce the expression of lincRNA-p21. Ectopic expression of lincRNA-p21 activated ER stress by inducing the expression of IRE1, CHOP, and GRP78 and up-regulating the phosphorylation level of PERK. Further research found that lincRNA-p21 induced-ER stress-mediated apoptosis is mainly mediated through reactive oxygen species (ROS) in hepatocellular carcinoma cells [132]. Collectively, lincRNA-p21 indirectly regulating ER stress has been demonstrated. However, how lincRNA-p21 activates ER stress remains to be further explored (Fig. 5a).

#### Invasion and metastasis

Invasion and metastasis are characteristics of malignant tumors and are leading causes of mortality. LncRNAs can also regulate these complex processes. Non-coding RNA activated by DNA damage (NORAD) has been found to be upregulated in numerous cancer tissues and involved in many biological processes of tumors, including migration and invasion [133–135]. In malignant melanoma, NORAD may act as a sponge for miR‐205 and reduce the transcriptional repression of the miR-205 target gene EGLN2, a key regulator of ER stress. NORAD silencing can inhibit UPR-related gene expression including that of GRP78, CHOP, and eIF2α. Downregulation of NORAD also restrained malignant melanoma cell migration and invasion. However, whether NORAD-induced ER stress promotes the invasion and metastasis of melanoma cells needs to be further studied [136]. In addition, lncRNA nuclear-enriched abundant transcript 1 (NEAT1) is newly discovered and has been localized in cell nuclear paraspeckles. Increasing evidence has revealed that NEAT1 is upregulated in multiple cancers and facilitates cell invasion and migration by being a sponge for miRNAs [137–140]. Recently, NEAT1 has been proven to be highly expressed in multiple myeloma, and knockdown of NEAT1 inhibited the invasion and metastasis of myeloma cells. Overexpression of NEAT1 promotes the expression of UPR-related proteins CHOP, XBP-1, and IRE1, while resveratrol attenuates the effects of NEAT1 on inducing ER stress [141]. Further understanding of the regulation of NEAT1 and UPR by resveratrol may provide a therapeutic strategy for cancer treatment. Another lncRNA, which has only been reported in colorectal cancer, is lncRNA Upregulated in Colorectal Cancer (LUCRC). The expression of LUCRC is increased in CRC tumor tissue and blood samples, and it is involved in functions such as tumorigenesis in vivo and CRC cell proliferation, migration, and invasion in vitro. Further study demonstrated that LUCRC can regulate the expression of the protein folding chaperone Bip and induce the splicing of XBP1 from XBP1u (unspliced) to XBP1s (spliced), resulting in activation of UPR to promote tumor progression [142] (Fig. 5b).

#### Tumor metabolism

The reprogramming of metabolism is a critical characteristic that supports the rapid proliferation of cancer cells. LncRNAs mediate glycolysis by regulating key enzymes in the pathway [143–145]. The lncRNA olfactory receptor family 3 subfamily A member 4 (OR3A4) is highly expressed in osteosarcoma and inversely related to the prognosis of patients. OR3A4 functions as a sponge for the miR-1207-5p modulated target gene Glucose-6-phosphate dehydrogenase (G6PD), an enzyme that catalyze the pentose phosphate pathway. Knockdown of OR3A4 increased the expression of miR-1207-5p and inhibited the expression of G6PD, blocking the pentose phosphate pathway and significantly reducing the level of nicotinamide adenine dinucleotide phosphate (NADPH). This abnormal metabolic pathway upregulated the redox state content, which increased the expression of PERK and IRE1 in osteosarcoma cells. The deletion of OR3A4 reduces NADPH production, which may lead to ROS accumulation and aggravate ER stress-induced apoptosis [145] (Fig. 5c).

### CircRNAs regulate the UPR in cancer

**Fig. 6:**
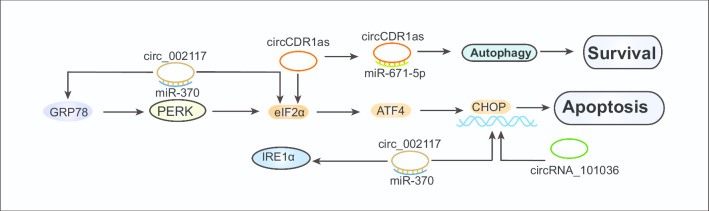
CircRNAs mediate tumor survival and apoptosis by UPR. Circ_002117 induced ER stress by upregulating GRP78, IRE1, eIF2α, and CHOP, subsequently resulting in the apoptosis of gastric cancer cells. CircCDR1as induces ER stress by upregulating eIF2α under hypoxia conditions, increasing OSCC cell survival. Moreover, circCDR1as promoted hypoxia-induced autophagy in OSCC cells by sponging miR-671-5p. CircRNA_101036 induced the UPR pro-apoptosis pathway by increasing CHOP protein, which induces apoptosis

As ncRNAs member, circRNAs have been reported to be expressed in almost all types of cells and possibly dysregulated in cancer [146]. CircRNAs are involved in tumor development and are becoming novel biomarkers for diagnosis and prognosis [147]. Recently, several studies have demonstrated that circRNAs could modulate cell survival through the UPR pathway. Circ_002117 was downregulated in gastric cancer compared with adjacent non-cancer tissues. The expression of circ_002117 was negatively correlated with the degree of malignant gastric cancer and positively correlated with the overall survival rate of patients. Augmented circ_002117 expression induced ER stress by upregulating the UPR pathway components GRP78, IRE1, eIF2α, and CHOP, subsequently resulting in apoptosis in gastric cancer cells. The underlying mechanism involves circ_002117 forming a sponge with miR-370, upregulating the HERPUD1 level, and facilitating ER stress-induced apoptosis [148]. CircCDR1as is highly expressed in OSCCs. Overexpression of circCDR1as induced ER stress by upregulating eIF2α under normal oxygen and hypoxia conditions, increasing OSCC cell viability [149]. Another study reported that circRNA_101036 was downregulated as a tumor suppressor gene in OSCC cell lines. Overexpression of circRNA_101036 induced the UPR pro-apoptosis pathway by increasing the levels of CHOP protein and ROS, which induces apoptosis [150]. Nevertheless, further investigations are still needed to elucidate the full picture of regulation of ER stress by circRNAs in cancer (Table 5, Fig. 6).

**Table 5:** CircRNAs regulate UPR pathway components

CircRNAs	Expression level	Tumor type	UPR-related Mechanism	Biological process	References
Circ_002117	Downregulated	Gastric cancer	Increases expression of GRP78, IRE1, eIF2α, and CHOP	Promotes apoptosis	[148]
CircCDR1	Upregulated	OSCC	Increases eIF2α expression	Inhibits apoptosis	[149]
CircRNA_101036	Downregulated	OSCC	Increases CHOP and ROS levels	Induces cell apoptosis	[150]

OSCC, oral squamous cell carcinoma

## Therapeutic strategies based on ncRNAs and the UPR in cancer

Chemotherapy usually fails because tumor cells acquire multidrug resistance, which is the result of multiple factors, including ER stress tolerance (ERST). Tunicamycin-induced ERST has been reported to be more resistant to cisplatin in lung cancer cells [151]. Some antitumor drugs are capable of stimulating GRP78 expression, which in turn induces cell resistance [152–154]. PERK activation can cause cell cycle arrest by specifically inhibiting the synthesis of certain cell cycle regulators. Cancer cells enter into quiescence, which is termed cancer cell dormancy [155, 156]. Dormant tumor cells are usually able to escape the toxic effects of chemotherapeutic drugs and are thought to be the cause of primary tumor progression and metastatic recurrence [157]. Previous studies have found that dormant tumor cells can upregulate all three major transducers of the UPR, IRE1α, PERK, and ATF6 [67, 158]. Moreover, GRP78 has previously been shown downstream of activated p38 and appears to play an important role in inducing drug resistance, which is involved in the maintenance of chemical resistance and stem cell populations in pancreatic cancer [152, 158, 159]. However, bortezomib-resistant myeloma cells did not upregulate GRP78. Instead, the low expression level of XBP1s and TP53 abnormalities were associated with bortezomib resistance [160]. In addition, ER stress has also been shown to be related to resistance to tyrosine kinase inhibitors by upregulating key survival signals, such as Bcl-xL [161]. Overall, UPR activation has been shown to mediate chemoresistance. Therefore, small molecule inhibitors targeting UPR components are promising candidates for overcoming drug resistance.

A growing number of studies have proven that miRNAs can act as tumor promoters or suppressors, and their dysregulation promotes tumor metastasis and therapeutic resistance by facilitating the activation of oncogenic signaling pathways [162–164]. For instance, miR-410 as a carcinogenic miRNA contributes to tumorigenesis and increases cell resistance to cisplatin in lung cell lines [165]. In contrast, miR-128 plays a role as a tumor suppressor that inhibits cancer stem cell self-renewal and increases A549 cell sensitivity to paclitaxel [166]. MiRNAs can also be dedicated to the modulation of UPR signaling pathways to regulate the therapeutic sensitivity of tumor cells. MiR-122 exhibited an inhibitory effect on the expression of a chaperone gene, and its overexpression leads to repression of the UPR pathway in HCC. Inhibition of miR-122 upregulates its target gene cyclin-dependent kinase 4 (CDK4) to enhance the stability of the 26S proteasome non-ATPase regulatory subunit 10 (PSMD10). This process activates the UPR to reduce the tumor cell apoptosis mediated by antitumor drugs [167].

CHOP is an important transcription factor involved in regulating apoptosis and drug sensitivity [168–170]. Studies have shown that the levels of CHOP mRNA and its protein level were significantly lower in lung cancer tissues compared with noncancerous tissues. CHOP downregulation predicted poor overall survival [171]. Furthermore, CHOP modulated the sensitivity of lung cancer cells to cisplatin through regulation of autophagy [171, 172]. MiR-146a directly targets the CHOP 3′UTR and downregulates CHOP expression, thus resulting in reduced sensitivity of lung cancer cells to cisplatin. MiR-146a may be a potential therapeutic target for resistant lung cancer [171]. Similarly, miR-1271 also directly acts on CHOP mRNA, and consequently promotes letrozole-resistance in breast cancer [173]. Other studies have demonstrated that miR-7112-3p is highly expressed in colorectal cancer tissues. However, sinoporphyrin sodium-induced photodynamic therapy (DVDMS-PDT) can downregulate miR-7112-3p in CX-1 cells, which directly acts on PERK and further regulates the PERK-ATF4-CHOP-Caspase3/8 signaling pathway, increasing DVDMS-PDT-induced cancer cell apoptosis [174].

GRP78 is highly expressed in 5-fluorouracil resistant cells, which can upregulate the expression of lncRNA myocardial infarction associated transcript (MIAT) by increasing OCT4. Inhibitors of GRP78 or MIAT can alleviate the drug resistance of tumor cells to 5-fluorouracil [175]. The potential role of UPR in regulating the transcription and function of lncRNAs was suggested. However, this requires further investigation to validate the mechanism of direct regulation.

## Conclusive remarks

Most of the existing evidence indicates that tumor cells initiate the UPR in response to major intrinsic changes and adverse environmental challenges where the UPR operates as a pro-oncogenic mechanism that drives several aspects of cancer development. Therefore, UPR modulators may be used as a biomarker of prognosis and a target of drug therapy. For instance, the expression level of XBP1s is significantly higher in numerous solid tumors, and its increased expression is associated with more malignant phenotypes and poor survival [42, 44, 176]. The overexpression of BiP is also associated with poor prognosis and weak response to treatment in clinical trials [176, 177]. Because the UPR can trigger pro-survival and pro-apoptotic signals, both inducers and inhibitors targeting UPR molecules can be used as therapeutic agents for tumors, including inhibitors of PERK (GSK2606414 and GSK2656157) [178, 179], ATF6 (16F16) [180], and IRE1α (MKC-3946, STF-083010) [181, 182], and a CHOP inducer (DK143) [183]. However, in certain circumstances, UPR downstream components have been shown to not only regulate ER stress-induced apoptosis, but also promote the growth of tumors. Thus, therapies targeting UPR components that promote cell death or survival require further detailed validation.

Under ER stress, tumor cells undergo a series of biological changes to adapt to growth, including ncRNA expression regulation. Conversely, ncRNAs also regulate UPR downstream target gene expression. UPR-related genes could be upstream regulators or downstream effectors of ncRNAs, forming an interaction network that jointly regulates the hallmarks of cancer. In this review, we systematically discussed mutual regulation of ER stress and ncRNAs (miRNAs, lncRNAs, and circRNAs) in the process of tumorigenesis and development. It is worth further exploring how the UPR is involved in communication and interaction between tumors and stromal cells and the regulation of angiogenesis and the immune response in the tumor microenvironment. Tumor cells releasing exosomes carrying miRNAs may play an important role in cell–cell functional interplay. Transmissible ER stress may be used as a way for cells to interact with each other in the tumor microenvironment. Extracellular vesicles serve as an interactive medium, containing proteins and ncRNAs, and should be further investigated. Over the years, there has been extensive evidence to support that ncRNAs can be selected as therapeutic targets, particularly in the field of oncology. Consequently, treatment targeting the lncRNA-miRNA-UPR pathway is an important strategy for cancer therapy, and the mechanism of action for many ncRNAs is consolidated in this framework. Therefore, significant improvements of cancer treatment are expected through targeting ncRNA and ER stress in the near future.

In conclusion, ncRNAs have been identified as major participants in complex UPR regulatory networks and have been found to be involved in many aspects of human malignancies. Additionally, the UPR also regulates ncRNA levels in tumor. This dual regulation of ER stress and ncRNAs provides further insights into the understanding of tumorigenesis and therapeutic strategies.

## Data Availability

The material supporting the conclusion of this review has been included within the article.

## Abbreviations

ER: Endoplasmic reticulum
ER stress: Endoplasmic reticulum stress
UPR: Unfolded protein responses
NcRNAs: Non-coding RNAs
ENCODE: Encyclopedia of DNA elements
MiRNAs: MicroRNAs
LncRNAs: Long non-coding RNAs
CircRNAs: Circular RNAs
UTR: Untranslated region
ERAD: Endoplasmic reticulum-associated degradation
OSCC: Oral squamous cell carcinoma
HCC: Hepatocellular carcinoma
TNBC: Triple-negative breast cancer
ERSE: Element or ER stress response element
AML: Acute myeloid leukemia
CRC: Colorectal cancer
SSD: *Spatholobus suberectus* Dunn
COM: *Cnidium officinale* Makino
NSCLC: Non-small cell lung cancer
EMT: Epithelial-to-mesenchymal transition
ECM: Extracellular matrix
MSCs: Mesenchymal stem cells
OPCs: Osteoblastic progenitor cells
ROS: Reactive oxygen species
NADPH: Nicotinamide adenine dinucleotide phosphate
ERST: ER stress tolerance
DVDMS-PDT: Sinoporphyrin sodium-induced photodynamic therapy
